# Effects of three exercise interventions on inhibitory control in college students with internet addiction: a functional near-infrared spectroscopy study

**DOI:** 10.3389/fpsyg.2025.1640967

**Published:** 2025-09-18

**Authors:** Zhimin Nie, Hainan Fan

**Affiliations:** Graduate School of Shandong Sport University, Shandong Sport University, Jinan, China

**Keywords:** internet addiction, exercise intervention, Footbike cycling, inhibitory control, functional near-infrared spectroscopy

## Abstract

**Background:**

Internet addiction (IA) poses a significant public health challenge, particularly among college students. Neurocognitive research points to dual inhibitory deficits as core mechanisms: impaired response inhibition drives impulsive loss of control, while deficient interference suppression heightens cue reactivity. While exercise shows potential for cognitive enhancement, its modality-specific effects on these distinct inhibitory subcomponents and underlying neurobiology remain unclear, hindering targeted interventions.

**Methods:**

This study employed a multi-modal intervention design with IA-diagnosed college students. Participants underwent supervised 8-week programs across three exercise modalities: Footbike, swimming, and basketball. Inhibitory control was assessed pre- and post-intervention using standardized cognitive tasks (Go/No-Go for response inhibition, Flanker for interference suppression), with concurrent neurofunctional monitoring via functional near-infrared spectroscopy (fNIRS) focusing on prefrontal subregions—dorsolateral prefrontal cortex (DLPFC), frontopolar cortex (FPC), and orbitofrontal cortex (OFC).

**Results:**

Footbike training demonstrated superior efficacy in enhancing inhibitory control compared to other modalities. It yielded significant improvements in both response inhibition (*d* = −1.67, 95% CI [−2.27, −1.07], *p* < 0.001) and interference inhibition (*d* = −0.78, 95% CI [−1.32, −0.25], *p* = 0.007), with neuroimaging revealing increased activation in associated regions including the DLPFC (*d* = 0.82, 95% CI [0.28, 1.35], *p* = 0.008) and FPC (*d* = 1.77, 95% CI [1.16, 2.38], *p* < 0.001). For interference inhibition function, basketball intervention showed significant improvement (*d* = −0.69, 95% CI [−1.22, −0.16], *p* = 0.005) and most strongly activated the OFC (*d* = −1.05, 95% CI [−1.06, −0.50], *p* = 0.004), though its effect on response inhibition was weaker. Swimming failed to demonstrate significant modality-specific benefits for any inhibitory domain. Distinct patterns of neural engagement across exercise types revealed dissociable neurocognitive pathways for inhibitory enhancement.

**Conclusion:**

Exercise modalities have distinct effects on IA-related inhibitory deficits: Footbike optimally enhances both subcomponents via DLPFC/FPC-mediated executive control, while basketball mainly engages OFC reward pathways with limited transfer. These findings provide a neurobiological basis for precision exercise prescriptions, identifying Footbike as optimal for dual inhibition deficits in IA. We propose a stratified framework using real-time fNIRS neurofeedback to match neurocognitive profiles with tailored exercise, advancing personalized interventions for addiction.

## 1 Introduction

While the internet’s exponential growth has revolutionized daily life and work efficiency, it has concurrently precipitated IA as a critical global public health challenge (Zhou et al., 2024). The China Statistical Report on Internet Development Status (Media Forum, 2025) reveals that the number of internet users in China has surged exponentially from 620,000 in 1997 to 1.108 billion by 2023, with the internet penetration rate reaching 78.6%. College students, characterized by ongoing neurocognitive and psychosocial maturation, heightened academic competition, emotional lability, and underdeveloped self-regulatory capacity, demonstrate heightened vulnerability to excessive internet use and addiction-like behaviors (Shi et al., 2025). Emerging empirical studies demonstrate that IA significantly impairs inhibitory control in adolescents (Salehi et al., 2022), triggering psychopathological symptoms (e.g., anxiety, depressive disorders), and precipitating functional decline in both academic performance and social adaptability (Fan et al., 2019). Therefore, investigating the neurocognitive mechanisms underlying IA among college students—particularly the inhibitory control-related neural substrates (e.g., impaired top-down cognitive regulation and hyper-reactivity to reward cues)—has emerged as a critical research priority to inform evidence-based interventions.

Research indicates that IA is strongly linked to inhibitory control deficits in college students (Kraplin et al., 2020). Exercise interventions, as a potential strategy to enhance inhibitory function, have shown promise in addressing IA-related cognitive impairments (Chen H. et al., 2023). Notably, mixed findings persist across studies: cycling training exhibits divergent outcomes in enhancing inhibitory control (Fan et al., 2021; Wang and Li, 2025), while running interventions similarly demonstrate inconsistent effects on cognitive inhibition (Nakutin and Gutierrez, 2019; Sandroff et al., 2016). Task-based behavioral evidence further elucidates this complexity: IA individuals displayed elevated error rates to no-go cues in the Go/No-Go task, indicating motor impulsivity dysregulation (Wang et al., 2020), while exhibiting prolonged reaction times in Flanker conflict tasks, suggesting impaired cognitive resource allocation mechanisms (Ma et al., 2025). Such discrepancies may stem from task-type heterogeneity, suggesting dual complexity in the modulatory effects of exercise interventions on inhibitory control—effects influenced not only by exercise modality (Zhang et al., 2023a) but also intricately tied to the measurement dimensions of cognitive assessment paradigms.

Task-specific heterogeneity reflects multidimensional cognitive regulation of inhibitory function, suggesting dissociable yet interactive neural mechanisms. Inhibitory control is primarily categorized into two subcomponents: response inhibition and interference inhibition (Zhang et al., 2023a), with distinct task designs prioritizing the assessment of domain-specific inhibitory dimensions. The interplay of dual inhibitory deficits creates a maladaptive cycle: compromised interference inhibition heightens attentional capture by task-irrelevant cues (Kang et al., 2021), and impaired response inhibition accelerates stimulus–response automatization. This cascade ultimately reduces Internet use to low-cognitive-load habitual behaviors, perpetuating addiction vulnerability.

Empirical studies have confirmed that the interaction of dual inhibitory deficits exacerbates IA (Zhang et al., 2024). Current exercise interventions predominantly target isolated inhibitory subcomponents, lacking systematic integration to disrupt the addiction cycle (Chen et al., 2020). Deciphering exercise modality-specific regulatory mechanisms on distinct inhibitory dimensions is critical to establishing a comprehensive multidimensional intervention framework. Notably, distinct exercise modalities differentially engage prefrontal subregions (Wang et al., 2023), which may serve as a neuroplasticity modulation entry point. Basketball training emphasizes team-based coordination (Li et al., 2020); swimming—a closed-skill, high-repetition activity (Arsoniadis et al., 2022); and Footbike training prioritizes dynamic balance control (Chow and Ha, 2024). These modalities engage distinct prefrontal cortex (PFC) mechanisms: Basketball recruits the PFC cognitive control network to simultaneously suppress motor impulsivity and dynamically adapt strategies, forming an execution-evaluation dual inhibitory loop (Brini et al., 2023; Luo et al., 2023). Swimming leverages PFC-mediated integration of response inhibition and interference suppression to optimize motor resource allocation and stabilize trajectory, an execution-monitoring bidirectional pathway (Serra et al., 2022); Footbike training, as an emerging modality, enhances dual inhibition via PFC activation by filtering irrelevant stimuli while prioritizing critical information processing, thereby creating a filtering-amplification synergy (Zhao et al., 2024).

The Footbike, originating from the wooden two-wheeled “Laufmaschine,” invented by German Karl Drais in the early 19th century, evolved into a structured fitness-oriented practice in Europe during the 1970s. Its inhibitory control-enhancing mechanisms stem from sustained cognitive challenges in dynamic environments and neuroplasticity reinforcement (Wang J. et al., 2024). Unlike basketball and swimming, Footbike training requires real-time processing of multimodal stimuli such as terrain navigation and balance adjustments (Evans et al., 2022; Vansteenkiste et al., 2014), forcing suppression of task-irrelevant actions for stability maintenance—a process optimizing PFC-mediated neuroplasticity (Wang Y. et al., 2024). Recent research demonstrates that open-skill sports surpass closed-skill training in enhancing inhibitory control (Li et al., 2024). This advantage stems from their dynamic environmental demands. Neurophysiological studies show dynamic balance training regulates inhibitory control through selective PFC activation (Menon and D'Esposito, 2022). While traditional open-skill activities (e.g., basketball) engage this mechanism (Veliks et al., 2024), cycling-based sports like Footbike impose greater demands on prefrontal inhibitory networks. This heightened neuromodulatory load arises from unique requirements for complex proprioception and spatial integration (Chang et al., 2020), involving simultaneous processing of gravitational adaptation and momentum control. We thus hypothesize that Footbike training exerts superior intervention efficacy on inhibitory control compared to conventional modalities, attributable to the heightened complexity of its dynamic balance training and the profound integration of proprioceptive input. This study holds dual practical significance: First, it elucidates the modality-specific alignment between exercise types and cognitive deficit profiles, providing a scientific basis for individualized intervention protocols; second, emerging modalities like Footbike training expand the potential to develop neuroregulatory interventions with high public accessibility, deepening the understanding of exercise-induced neurobiological mechanisms and advancing the construction of multimodal cognitive rehabilitation frameworks.

The differential efficacy of intervention protocols arises from subregion-specific activation patterns within the PFC. As the brain’s “executive hub” for higher-order cognition, the PFC exhibits neuroplasticity directly linked to the regulation of complex behaviors (Friedman and Robbins, 2022; Kang et al., 2022). Empirical evidence reveals that dual inhibitory mechanisms—interference inhibition and response inhibition—shape prefrontal functional dynamics through distinct neural circuits: Interference inhibition enhances filtering efficiency via strengthened attentional control networks (van Moorselaar and Slagter, 2020), whereas response inhibition achieves adaptive behavioral calibration by suppressing prepotent responses (Friehs et al., 2021). Their synergistic interplay amplifies prefrontal efficiency and impulse control. The neurobiological underpinnings of these dual inhibitory mechanisms provide quantifiable intervention targets for impulse control disorders such as IA (Zhang et al., 2023b). Functional near-infrared spectroscopy (fNIRS) enables real-time monitoring of prefrontal cortex activity, providing scientific evidence to establish sequential causal pathways linking inhibition types—specifically interference and response inhibition—to neural plasticity in the prefrontal cortex and subsequent behavioral change. This study employs fNIRS to investigate how distinct exercise intervention protocols modulate domain-specific activation patterns in the prefrontal cortex, thereby regulating these cognitive control mechanisms. The findings aim to deliver targeted intervention strategies for impulse control disorders, exemplified by internet addiction, ultimately optimizing personalized exercise-based interventions.

## 2 Materials and methods

### 2.1 Study participants

Using G*Power 3.1 (Faul et al., 2007), *a priori* power analysis for a 4 × 2 repeated-measures ANOVA indicated that 76 participants would provide 95% power (*f* = 0.25, α = 0.05). To account for potential attrition, 120 eligible undergraduates (aged 18–22 years) were recruited through campus bulletin board postings at Shandong Sport University in February 2025. Participants underwent block randomization stratified exclusively by baseline Internet Addiction Test (IAT; Young, 1998) scores according to predefined severity strata: Stratum 1 (50–59, moderate-low), Stratum 2 (60–69, moderate-high), and Stratum 3 (70–79, severe). Within each stratum, an independent statistician generated separate randomization sequences using SPSS 28.0 with a fixed block size of 8. This ensured balanced allocation with exactly two participants assigned to each of the four groups (Footbike Training, Basketball Training, Swimming Training, Control) per randomization block. Allocation was implemented via sequentially numbered, opaque, sealed envelopes opened after baseline assessment completion. This procedure yielded balanced group assignment (*n* = 30 per group). Baseline equivalence was confirmed through statistical testing, with characteristics detailed in Table 1.

**Table 1:** Baseline characteristics of participants across the four experimental groups.

Characteristics	Swimming group	Basketball group	Footbike group	Control group	*F*	*P*
Age (years)	20.97 ± 1.10	21.13 ± 0.94	20.63 ± 1.19	21.10 ± 0.88	1.46	0.229
Height (cm)	172.05 ± 8.05	173.82 ± 7.49	172.09 ± 7.98	173.32 ± 6.74	0.41	0.745
Body Mass (kg)	73.66 ± 5.51	71.53 ± 5.09	71.16 ± 5.04	72.77 ± 5.30	1.44	0.235
PARS	26.90 ± 1.09	26.80 ± 1.19	27.47 ± 1.11	26.93 ± 1.39	1.88	0.138
IAT	64.90 ± 2.57	64.83 ± 2.31	65.73 ± 2.80	65.57 ± 2.22	1.02	0.388

Data presented as mean ± SEM. Group comparisons performed using one-way ANOVA; all *p* > 0.05 indicate no significant baseline differences across groups.

The inclusion criteria were as follows: (a) IAT scores 50–79 (moderate-to-severe symptoms), (b) health criteria (normal/corrected vision per Snellen chart, no color blindness per Ishihara test, right-handedness > +40 on Edinburgh Inventory, no neuropsychiatric disorders), and (c) Physical Activity Rating Scale (PARS; Fu et al., 2023) scores 5–42 (moderate exercise); The study protocol was reviewed and approved by the Ethical Committee of Shandong Sport University (#2024066) and was in accordance with the Declaration of Helsinki.

### 2.2 Intervention protocol

The 8-week intervention program consisted of three exercise modalities (basketball, swimming, and Footbike), with each session lasting 45 min and administered twice weekly.

Exercise intensity was standardized using the Karvonen method (Niwa et al., 2022; Sansare et al., 2021), calculated as follows: Target heart rate = Resting HR + (HRR × Intensity%), where HRR = HRmax - Resting HR and HRmax = 207–0.7 × age. Moderate intensity corresponded to 55–65% of HRmax. Exercise intensity was monitored using a Polar M430 heart rate monitor (Polar Electro Oy, Finland). Adherence to the prescribed intensity was verified in 100% of sessions, with deviations >5% prompting immediate adjustments by trained staff.

Basketball training included dribbling, shooting drills, and team-tactical exercises. The swimming group practiced multiple swimming strokes to enhance endurance and speed, while the Footbike group focused on balance control, curved-path maneuvers, and high-velocity propulsion techniques. All sessions were conducted under the supervision of professional coaches, with adjustments made based on participants’ physical condition and progress. A no-exercise control group was established to maintain their usual lifestyle habits without additional physical training.

### 2.3 Measurement of inhibitory control

This study employed the Go/No-Go task (Beerten-Duijkers et al., 2021; Wu et al., 2024) and the Flanker task (Mikneviciute et al., 2023) to measure response inhibition and interference control, respectively. The experiment was conducted using E-Prime 3.0 software (Psychology Software Tools, Inc.), with participants seated approximately 60 cm from a computer screen where all stimuli were presented. Prior to the formal trials, a standardized verbal briefing was administered to ensure task comprehension. None of the participants reported prior experience with cognitive inhibition paradigms. Stimulus presentation and response collection were implemented with temporal resolution controlled to ± 1 ms. Inhibitory efficiency was quantified using inverse scoring, such that reduced values reflected enhanced cognitive control capacity.

**Figure 1:**
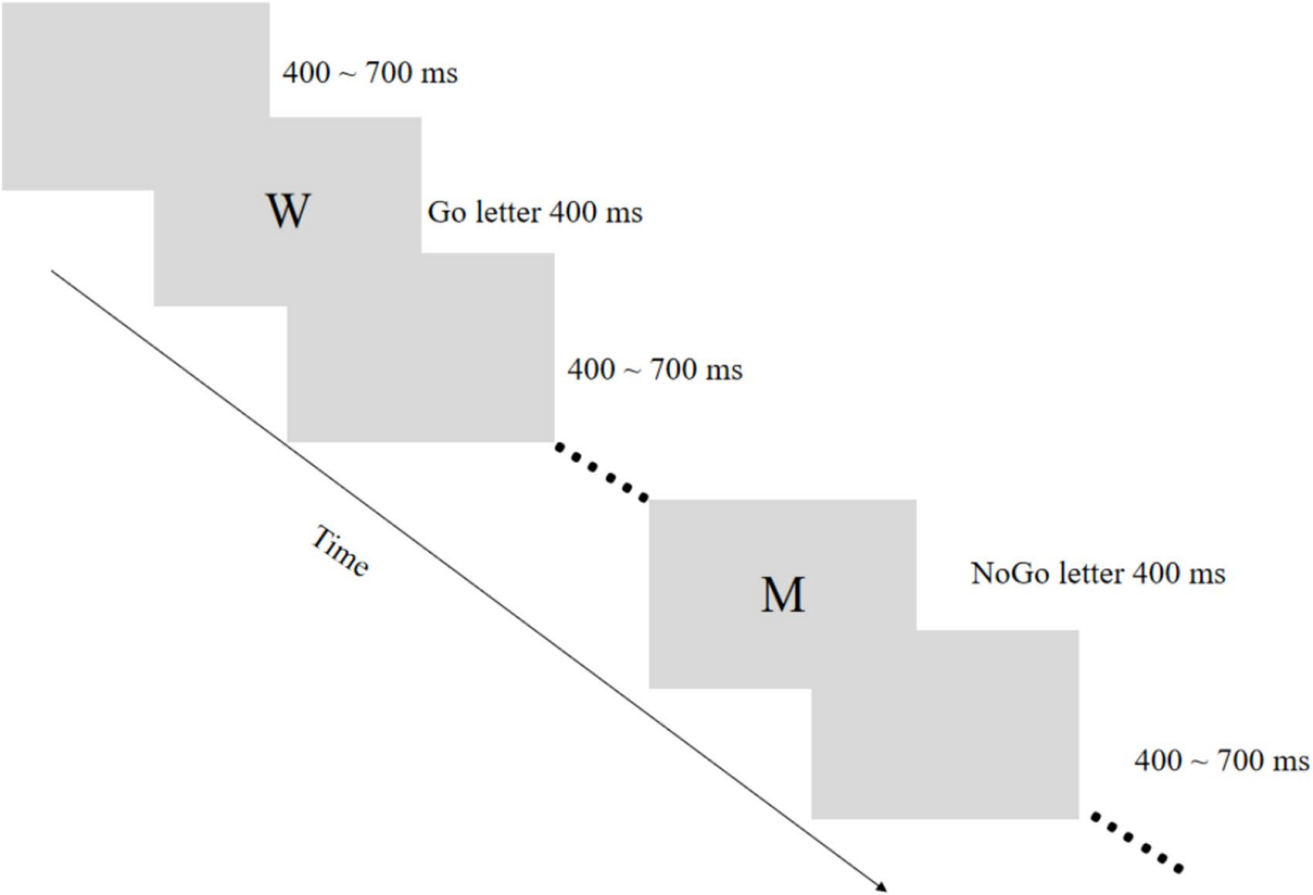
Go/No-Go task procedure for response inhibition assessment.

For the Go/No-Go task, participants were instructed that M represented the Go stimulus and W the NoGo stimulus. Appearance of M required pressing the “J” key with the index finger as rapidly and accurately as possible, while W required no responses. The primary dependent variable was No-Go accuracy (correct inhibition rate), with additional measures including Go trial reaction time and accuracy recorded (Figure 1).

**Figure 2:**
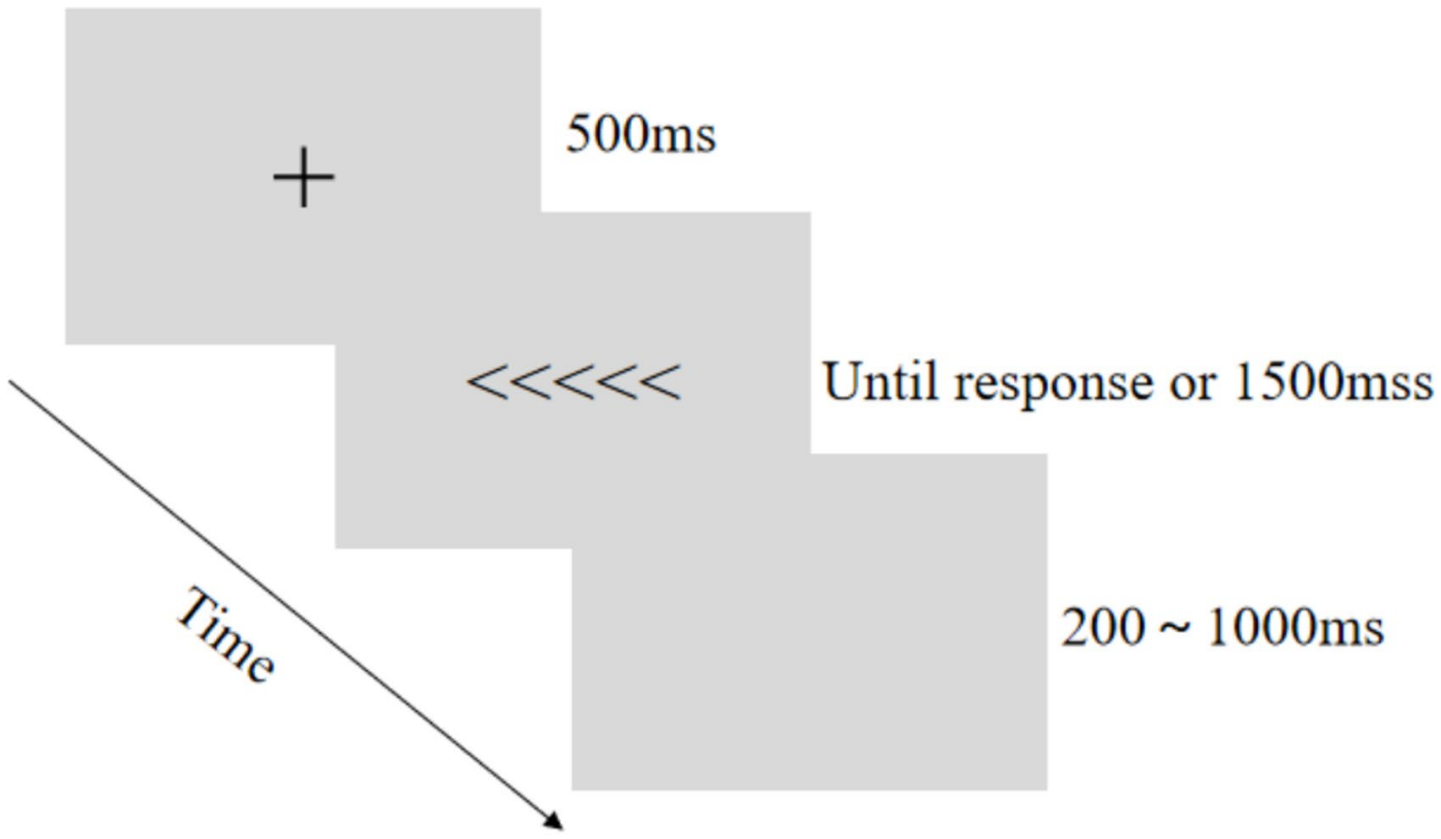
Flanker task procedure for inhibitory control assessment.

In the Flanker task, each trial began with a 500 ms central fixation cross (“+”), followed by horizontally arranged arrow stimuli where the central arrow served as the target flanked by distractors. Trials were classified as consistent (all arrows identical: > > > > > or < < < < <) or inconsistent (incongruent flankers: > > < > > or < < > < <) based on target-distractor congruence. Participants identified the central target’s direction (pressing “F” for < or “J” for >) as rapidly and accurately as possible. Trials terminated upon response or after 1,500 ms, followed by a 200–1,000 ms inter-trial interval (Figure 2).

Both the Go/No-Go and Flanker tasks comprised practice and formal experimental phases, with participants required to achieve at least 90% accuracy during practice trials before proceeding to formal testing. The Go/No-Go task consisted of 4 blocks containing 240 stimuli (25% No-Go trials), while the Flanker task included 120 experimental trials (50% congruent/incongruent conditions) presented in randomized order.

### 2.4 FNIRS data collection

Data acquisition was performed in a dedicated electromagnetic-shielded chamber (ambient noise < 40 dB, illumination 50 ± 5 lux) to minimize interference from acoustic disturbances and photon scattering artifacts during fNIRS recordings. In the present study, hemodynamic signals in localized brain regions during an inhibitory control task were acquired using a portable fNIRS system (LIGHTNIRS, Japan). The fNIRS system utilized three wavelengths (780 nm, 805 nm, 830 nm) with a sampling rate of 13.33 Hz. An 8 × 8 optode configuration was implemented, comprising 22 measurement channels (CH1-CH22) through an alternating emitter-detector arrangement at 30 mm inter-optode spacing. Optodes were positioned over prefrontal regions according to the international 10–20 system for EEG electrode placement. Channel coordinates were acquired using a 3D digitizer (FASTRAK system, Polhemus, United States) and coregistered to Montreal Neurological Institute (MNI) standard space through SPM12 computational routines. Anatomical localization of measurement channels was achieved by combining Brodmann area parcellation with structural landmark identification.

**Figure 3:**
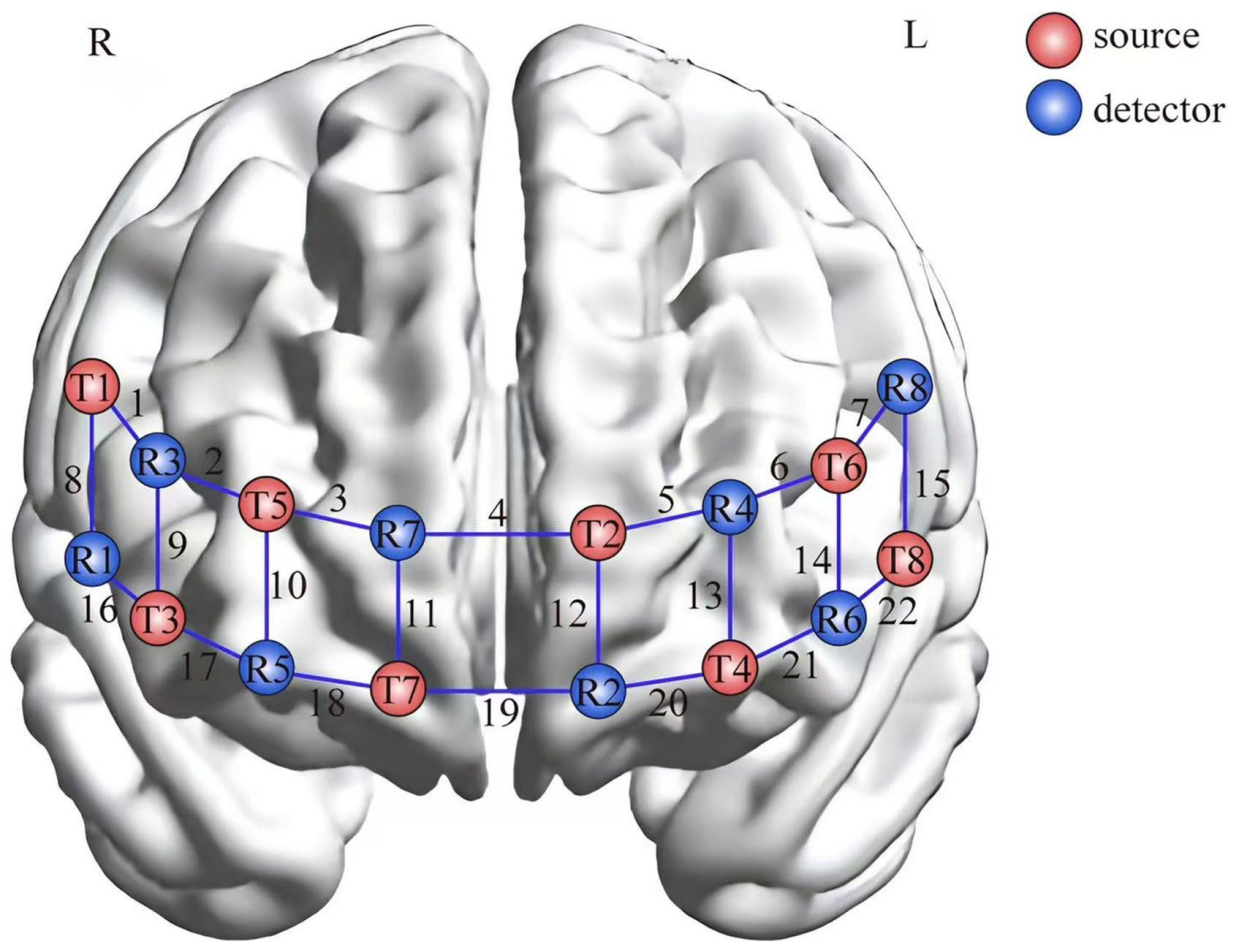
Schematic representation of fNIRS optode array configuration.

Hemodynamic changes in prefrontal subregions were analyzed through channel-wise signal aggregation: DLPFC oxygenation was derived from averaged signals in CH1, CH7, CH15, and CH22; FPC activation was calculated as the mean of CH2-6, CH9-14, CH17, and CH18; Inferior frontal cortex (IFC) responses were directly obtained from CH16; OFC signals were aggregated from CH19-21 (Figure 3).

### 2.5 Statistical analysis

Hemodynamic data underwent preprocessing via MATLAB (2013b) and Homer2 toolboxes, implementing signal conversion, artifact correction, and bandpass filtering (0.01–0.2 Hz) for noise attenuation, with oxygenated hemoglobin (HbO) concentration changes serving as the primary analytical metric.

Statistical analysis using SPSS 28.0 employed a 2 (Time: Pretest, Posttest) × 4 (Group: Footbike Training, Basketball Training, Swimming Training, Control) repeated-measures ANOVA was conducted. Given the within-subjects Time factor comprised only two levels, sphericity assumptions were inherently satisfied and thus not formally tested. Should a significant main effect of Group emerge, Bonferroni-adjusted pairwise comparisons would be performed regardless of interaction significance, with adjusted α = 0.0083 (0.05/6 comparisons). Upon identification of significant interaction effects, simple effect analyses were performed: (a) Time effects were examined within each Group to assess Pre-Post differences, and (b) Group effects were evaluated separately at each Time point (Pretest/Posttest) for between-group comparisons, with Bonferroni correction controlling family-wise error rates across all pairwise comparisons (adjusted α = 0.0125 per time point). Effect sizes were reported as partial eta-squared ($\eta_{p}^{2}$) for ANOVA effects and Cohen’s *d* for pairwise contrasts.

## 3 Results

### 3.1 Exercise modalities effects on response inhibition in IA

#### 3.1.1 Go/No-Go task behavioral results

Repeated-measures ANOVA on No-go accuracy rates revealed a significant main effect of time (*F*_(1, 116)_ = 51.199, *p* = 0.001, $\eta_{p}^{2}$= 0.353) and a significant time × exercise type interaction (*F*_(3, 116)_ = 2.776, *p* = 0.046, $\eta_{p}^{2}$ = 0.081). The main effect of exercise type was non-significant (*F*_(3, 116)_ = 2.228, *p* = 0.090, $\eta_{p}^{2}$= 0.066). Simple effects analyses revealed no significant pre-intervention differences in No-go accuracy among groups (*p* = 0.985). Following the intervention, significant differences emerged between the Control group and the Footbike group (*p* < 0.001), basketball group (*p* = 0.001), and swimming group (*p* < 0.001); whereas no significant differences were observed for Footbike vs. basketball (*p* = 0.399), Footbike vs. swimming (*p* = 0.921), or basketball vs. swimming (*p* = 0.472).

All exercise groups demonstrated significant improvements in No-go accuracy post-intervention versus baseline. Effect size analysis (Cohen’s *d*, calculated as [baseline - post-intervention] /SD_pooled_) revealed substantially greater enhancement in the Footbike group (*d* = −1.67, 95% CI [−2.27, −1.07]) compared to the swimming group (*d* = −1.61, 95% CI [−2.20, −1.02]), basketball group (*d* = −1.07, 95% CI [−1.62, −0.52]), and control group (*d* = −0.26, 95% CI [−0.78, 0.26]). The control group’s confidence interval spanning zero indicated statistically non-significant improvement (Table 2).

**Table 2:** Response inhibition performance across exercise modalities: pre- vs. post-intervention.

Measure	Time	Swimming group	Basketball group	Footbike group	Control group
No-go accuracy (%)	Pre	91.71 ± 0.06	92.25 ± 0.07	91.58 ± 0.06	91.92 ± 0.07
	Post	98.91 ± 0.02	98.00 ± 0.03	99.04 ± 0.02	93.75 ± 0.07
	*F*	20.23	14.02	24.48	1.35
	*P*	**<0.001**	**<0.001**	**<0.001**	0.248
	Cohen’s *d*	−1.61	−1.07	−1.67	−0.26
	95% CI	[−2.20, −1.02]	[−1.62, −0.52]	[−2.27, −1.07]	[−0.78, 0.26]
DLPFC HbO	Pre	5.09 ± 8.05	2.60 ± 7.98	0.95 ± 6.22	2.10 ± 7.03
	Post	−3.42 ± 6.46	−0.88 ± 5.93	−3.40 ± 4.24	0.90 ± 7.66
	*F*	24.75	4.49	7.31	0.51
	*P*	**<0.001**	**0.037**	**0.008**	0.475
	Cohen’s d	1.17	0.50	0.82	0.16
	95% CI	[0.61, 1.72]	[−0.03, 1.02]	[0.28, 1.35]	[−0.35, 0.68]
FPC HbO	Pre	5.57 ± 7.05	6.08 ± 6.91	6.57 ± 6.42	2.61 ± 6.12
	Post	−3.21 ± 6.30	−3.14 ± 6.24	−3.31 ± 4.59	−0.47 ± 7.85
	*F*	23.35	27.95	33.34	3.00
	*P*	**<0.001**	**<0.001**	**<0.001**	0.086
	Cohen’s d	1.31	1.40	1.77	0.44
	95% CI	[0.74, 1.88]	[0.82, 1.98]	[1.16, 2.38]	[−0.08, 0.96]
IFC HbO	Pre	1.31 ± 6.68	3.07 ± 7.15	1.16 ± 6.61	1.62 ± 5.01
	Post	−4.76 ± 9.00	−3.47 ± 8.26	0.75 ± 7.86	−2.79 ± 6.05
OFC HbO	Pre	−0.17 ± 5.02	2.88 ± 4.90	2.04 ± 4.83	2.64 ± 7.89
	Post	−2.63 ± 7.64	−1.40 ± 5.0.66	−0.55 ± 5.23	−0.04 ± 6.32

Data presented as mean ± SEM. Cohen’s d effect sizes calculated for within-group pre-post comparisons. Bolded *p*-values indicate significance (*p* < 0.05). HbO: oxygenated hemoglobin (units: Δμmol/L).

Collectively, Footbike, basketball, and swimming interventions significantly improved No-go accuracy in college students with IA, with Footbike exhibiting the most pronounced enhancement in inhibitory control.

#### 3.1.2 Go/No-Go task fNIRS results

Repeated-measures ANOVA was conducted on HbO concentration changes across brain regions, with exercise type (Control group, basketball, swimming, Footbike) as the between-subjects factor and time (pre−/post-intervention) as the within-subjects factor during Go/No-Go task performance. For the DLPFC, analyses revealed a significant main effect of time (*F*_(1, 116)_ = 27.93, *p* < 0.001, $\eta_{p}^{2}\;$= 0.23), a significant time × exercise type interaction (*F*_(3, 116)_ = 23.25, *p* = 0.025, $\eta_{p}^{2}$ = 0.09), and a non-significant main effect of exercise type (*F*_(3, 116)_ = 1.23, *p* = 0.303, $\eta_{p}^{2}\;$= 0.04). For the FPC, significant main effects of time (*F*_(1, 116)_ = 77.09, *p* = 0.001, $\eta_{p}^{2}$ = 0.45) and time × exercise type interactions (*F*_(3, 116)_ = 3.15, *p* = 0.029, $\eta_{p}^{2}$ = 0.09) were observed, while the main effect of exercise type was non-significant (*F*_(3, 116)_ = 0.07, *p* = 0.975, $\eta_{p}^{2}$ = 0.02). For the IFC and OFC, significant main effects of time were observed, while *post hoc* analyses of DLPFC data showed no significant between-group differences in HbO concentration at baseline (*p* = 0.261). Post-intervention, the control group exhibited significantly lower activation compared with the Footbike (*p* = 0.015) and swimming groups (*p* = 0.018), but not the basketball group (*p* = 0.314). No significant differences emerged among exercise groups: Footbike vs. basketball (*p* = 0.147), Footbike vs. swimming (*p* = 0.990), or basketball vs. swimming (*p* = 0.156). Other main effects and interactions remained non-significant. HbO concentration changes in the DLPFC across groups revealed significant pre-to-post intervention differences in the Footbike (*p* = 0.008), basketball (*p* = 0.037), and swimming (*p* < 0.001), but not the control group (*p* = 0.475). Effect size comparisons (Cohen’s *d*) indicated varying magnitudes of improvement across groups: the swimming group demonstrated the most pronounced enhancement (*d* = 1.17, 95% CI [0.61, 1.72]), followed by the Footbike group (*d* = 0.82, 95% CI [0.28, 1.35]), and basketball group (*d* = 0.50, 95% CI [−0.03, 1.02]). The control group showed a non-statistically significant effect (*d* = 0.16, 95% CI [−0.35, 0.68]).

Simple effects analyses stemming from the significant time × exercise type interaction revealed no between-group differences in FPC HbO concentrations at pre-intervention (*p* = 0.162), but significant between-group differences emerged at post-intervention (*p* = 0.035). Within-group analyses demonstrated substantial pre-post improvements in the exercise groups: Footbike (*d* = 1.77, 95% CI [1.16, 2.38], *p* < 0.001), basketball (*d* = 1.40, 95% CI [0.82, 1.98], p < 0.001), and swimming (*d* = 1.31, 95% CI [0.74, 1.88], *p* < 0.001), while the control group exhibited no significant change (*d* = 0.44, 95% CI [−0.08, 0.96], *p* = 0.086).

Collectively, exercise interventions were associated with enhanced neural activation in the DLPFC and FPC during Go/No-Go task among college students with IA. Among exercise modalities, Footbike training elicited the most pronounced activation increases in these prefrontal regions.

### 3.2 Exercise modalities effects on interference inhibition in IA

#### 3.2.1 Conflict effects: behavioral results

Conflict effects were calculated as the reaction time difference between congruent and incongruent trials, consistent with Yin Hengchan’s methodology (Yin et al., 2014). Repeated-measures ANOVA was applied to these conflict effect values. Significant main effects of time (*F*_(1, 116)_ = 8.20, *p* = 0.005, $\eta_{p}^{2}$ = 0.08) and time × exercise type interactions (*F*_(3, 116)_ = 3.01, *p* = 0.034, $\eta_{p}^{2}$ = 0.10) emerged for conflict effects (RT differences: incongruent minus congruent trials), while exercise type main effects were non-significant (*F*_(3, 116)_ = 0.37, *p* = 0.772, $\eta_{p}^{2}$ = 0.01). *Post hoc* simple effects analyses revealed no significant between-group differences in incongruent trial reaction times at baseline (*p* = 0.576). Post-intervention, the control group showed significantly longer reaction times compared with both the Footbike (*p* = 0.039) and basketball groups (*p* = 0.026) (Table 3).

**Table 3:** Interference inhibition performance and cortical activation across exercise modalities pre- and post-intervention.

Measure	Time	Swimming group	Basketball group	Footbike group	Control group
Conflict effect (ms)	Pre	−62.2 ± 59.53	−63.4 ± 59.21	−63.4 ± 57.66	−44.0 ± 54.16
	Post	−46.6 ± 43.77	−22.6 ± 58.89	−25.6 ± 35.92	−56.6 ± 66.11
	*F*	1.13	8.37	7.49	0.384
	*P*	0.291	**0.005**	**0.007**	0.770
	Cohen’s d	−0.30	−0.69	−0.78	0.21
	95% CI	[−0.82, 0.22]	[−1.22, −0.16]	[−1.32, −0.25]	[−0.31, 0.73]
OFC HbO	Pre	0.63 ± 13.40	−6.48 ± 10.46	−6.41 ± 8.72	0.43 ± 16.15
	Post	1.76 ± 12.71	4.70 ± 10.72	2.17 ± 11.89	−1.93 ± 13.48
	*F*	0.08	8.74	5.36	0.37
	*P*	0.776	**0.004**	**0.023**	0.543
	Cohen’s d	−0.09	−1.05	−0.82	0.16
	95% CI	[−0.60, 0.43]	[−1.06, −0.50]	[−1.36, −0.29]	[−0.36, 0.68]
DLPFC HbO	Pre	−1.87 ± 9.05	−3.32 ± 8.39	−3.42 ± 9.68	−1.64 ± 6.86
	Post	1.07 ± 9.77	3.16 ± 8.25	2.98 ± 6.71	−0.73 ± 4.70
IFC HbO	Pre	0.64 ± 10.45	0.08 ± 8.67	0.49 ± 8.10	0.94 ± 8.75
	Post	−0.29 ± 9.67	1.70 ± 9.85	1.36 ± 7.13	−1.50 ± 8.54
FPC HbO	Pre	1.02 ± 6.81	−2.55 ± 5.75	−0.22 ± 8.81	−0.26 ± 10.17
	Post	1.39 ± 5.07	4.87 ± 6.01	0.08 ± 5.65	−0.92 ± 8.28

Data presented as mean ± SEM. Cohen’s d effect sizes calculated for within-group pre-post comparisons. Bolded *p*-values indicate significance (*p* < 0.05). HbO: oxygenated hemoglobin (units: Δμmol/L).

Longitudinal changes in conflict effect reaction time differences revealed significant improvements in the Footbike (*d* = −0.78, 95% CI [−1.32, −0.25], *p* = 0.007) and basketball groups (*d* = −0.69, 95% CI [−1.22, −0.16], *p* = 0.005), but non-significant changes in swimming (*p* = 0.291) and control groups (*p* = 0.385). Effect size comparisons confirmed Footbike’s superior efficacy over basketball.

#### 3.2.2 Conflict effects: fNIRS results

Prefrontal HbO concentration differences during Flanker task performance were analyzed across exercise groups. Repeated-measures ANOVA on post-intervention HbO concentrations during Flanker task performance revealed a significant main effect of time in the OFC (*F*_(1, 116)_ = 5.87, *p* = 0.017, $\eta_{p}^{2}\;$= 0.06) and a significant time × exercise type interaction (*F*_(3, 116)_ = 2.73, *p* = 0.048, $\eta_{p}^{2}\;$= 0.08). All other main effects and interactions remained non-significant (*F*_(3, 116)_ = 0.73, *p* = 0.538, $\eta_{p}^{2}\;$= 0.02). The main effect of test time was significant in the DLPFC (*F*_(1, 116)_ = 12.87, *p* = 0.001, $\eta_{p}^{2}$= 0.12). Neither the test time × exercise type interaction (*F*_(3, 116)_ = 1.39, *p* = 0.252, $\eta_{p}^{2}$= 0.04) nor the main effect of exercise type (*F*_(3, 116)_ = 0.19, *p* = 0.906, $\eta_{p}^{2}$= 0.01) was significant; no other significant effects were observed.

*Post hoc* simple effects analyses revealed no significant between-group differences in HbO concentrations of OFC data before or after the intervention. Longitudinal OFC HbO changes showed significant improvement in the basketball group, whereas the Footbike group and other groups exhibited no significant changes. Effect size comparisons confirmed the significantly superior efficacy of the basketball group.

## 4 Discussion

### 4.1 Neurocognitive mechanisms of response inhibition enhancement

Our findings demonstrate that Footbike, basketball, and swimming interventions effectively improved response inhibition in college students with IA, consistent with established evidence linking exercise to enhanced inhibitory control (Yun et al., 2025). Further research found that Footbike training exhibits unique advantages in enhancing response inhibition.

Footbike propulsion relies on rhythmic leg-driven thrusting motions, during which athletes maintain a swallow-like balance posture akin to gymnastics beam techniques to stabilize during free-gliding phases (Symeonidou et al., 2023). Dynamic balance maintenance demands precise postural control through coordinated activation of core trunk and lower limb muscles, forming a stable kinetic chain to ensure body stabilization. Footbike’s unique motion pattern compels athletes to continuously adjust their center of gravity during dynamic movement and refine postural control via proprioceptive feedback. Such balance training enhances neuromuscular precision in the central nervous system (Jor et al., 2025), demonstrating superior efficacy in dynamic balance and motor coordination training. In contrast, basketball training effectively enhances reaction speed and vertical jump performance (Jiang and Xu, 2022; Sugiyama et al., 2021), yet imposes lower demands on dynamic balance and motor coordination. Swimming primarily targets muscle strength development and hypertrophy across major muscle groups (de Azambuja et al., 2021; Dopsaj et al., 2020), exerting limited direct effects on response inhibition enhancement. Footbike training demonstrates superior efficacy in enhancing response inhibition by strengthening multisensory integration (vestibular-proprioceptive-visual systems), suggesting enhanced neural pathways for inhibitory control (Kwag and Zijlstra, 2022; Rowley et al., 2022).

Empirical studies confirm that Footbike training effectively enhances FPC activation during cognitive tasks (Chen et al., 2024; Zheng et al., 2022). As the supreme integrative hub of the prefrontal cortex, the FPC orchestrates complex decision-making and regulates goal-directed behaviors. This is achieved through multimodal information integration and dynamic resource allocation mechanisms (Chen Y. et al., 2023). Footbike’s dynamic balance demands-requiring real-time postural adjustments in three-dimensional space-robustly activate FPC neural networks through high-load multitasking processing. This is evidenced by FPC-mediated suppression of prepotent responses and optimized behavioral selection, leading to reduced reaction times when responding to sudden interference (Shao et al., 2023). During free-gliding phases, athletes must sustain a multicomponent balance posture involving single-leg support, contralateral leg extension, and forward trunk inclination. This process enhances dynamic neuromuscular control through closed-loop regulation of proprioceptive input and motor output (Muehlbauer, 2021; Papalia et al., 2020). Such improvement relies not only on adaptive reorganization of peripheral motor systems but is also closely associated with FPC neural circuit activation and cross-regional neural collaboration (Sathe et al., 2024; Xu et al., 2024a). Neuroimaging studies reveal that balance training differentially activates the PFC (Rubega et al., 2021). Enhanced FPC activation is directly linked to postural stability maintenance, rapid interference response, and improved motor precision (Pan and Tang, 2025).

Swimming elicits stronger DLPFC activation compared to Footbike training. This is attributable to its dual cognitive demands on resource allocation and motor-respiratory coordination. Swimming, as a whole-body multi-joint coordination task (e.g., spatiotemporal precision in freestyle arm strokes, leg kicks, and trunk rotation) (Silva et al., 2020). These demands drive deeper DLPFC engagement in movement coordination (Rubega et al., 2021; Xu et al., 2024b). Conversely, aquatic environments require continuous multisensory integration. This necessity arises from their high resistance and buoyancy properties (Watanabe et al., 2020). Tactile input from water currents enhances spatial perception. Simultaneously, buoyancy-induced demands for dynamic postural adjustments amplify DLPFC activation during proprioception and balance regulation. In contrast, Footbike training primarily involves repetitive lower-limb pedaling with fixed kinematic patterns. It imposes lower demands on whole-body neuromuscular coordination and higher-order cognitive engagement (Liu et al., 2023). Additionally, it lacks aquatic environments’ complex multisensory integration and real-time adaptive challenges. Consequently, swimming demonstrates superior efficacy in promoting DLPFC activation compared to Footbike training. Although DLPFC exhibits neural activation, its functional activity is not reflected in behavioral metrics. This discrepancy may stem from partial neural resources being diverted to proprioceptive processing and postural balance regulation — neural activities not directly linked to cognitive inhibition.

In summary, Footbike training demonstrates superior efficacy for improving dynamic balance and motor coordination. It elicits robust neural activation in brain regions associated with response inhibition, which leads to marked enhancements in inhibitory control capabilities. This finding provides novel perspectives and empirical support for enhancing response inhibition in college students with IA through exercise-based interventions.

### 4.2 Neurocognitive mechanisms of interference inhibition enhancement

This study revealed that both basketball and Footbike training improved interference inhibition in college students with IA, with Footbike demonstrating superior efficacy in this regard.

Footbike training, by demanding high rhythmic precision, postural stability, and adaptive responses to sudden contextual changes, compels athletes to continuously filter task-irrelevant stimuli and prioritize goal-directed attention (Li et al., 2025), thereby enhancing interference inhibition and cognitive control via prefrontal-mediated neuroplasticity (Macoun et al., 2021). Footbike athletes require precise postural control to manage environmental perturbations (e.g., wind gusts, track irregularities). This necessitates pre-activation of brain regions for higher-order cognition—a mechanism reinforcing interference inhibition through neural circuit potentiation (Yu et al., 2021; Zhao et al., 2020). Empirical studies indicate that task complexity significantly modulates cognitive load (Madinabeitia-Cabrera et al., 2023). Footbike’s unique biomechanical characteristics contribute to its superior efficacy in enhancing interference inhibition. In contrast, basketball—despite its fast-paced and contextually dynamic nature-diverts attentional resources from sustained focus due to high demands on team coordination and rapid decision-making (Aivaz and Teodorescu, 2022). High team coordination demands limit training of sustained attentional focus, reducing efficacy in interference inhibition enhancement. Swimming occurs in closed-skill environments with minimal external interference (Kim et al., 2021). It demonstrates weaker efficacy in training interference resistance, due to lacking continuous interference-filtering demands. Footbike’s dynamic environments provide critical interference-filtering demands essential for cognitive inhibition enhancement. In summary, Footbike training demonstrates superior efficacy in enhancing interference inhibition, attributable to its unique biomechanical characteristics and high attentional demands that prioritize sustained cognitive engagement during dynamic balance challenges.

Compared to Footbike training, basketball elicits more pronounced OFC activation. This neural modulation effect stems from basketball’s open-skill team dynamics (Tsai et al., 2022). These dynamics require real-time integration of multidimensional information (e.g., teammate positioning, opponent defense anticipation, score pressure). They also involve rapid cost–benefit evaluation during high-risk decisions (e.g., weighing interception risks against scoring opportunities) (Yang et al., 2021). Such socially complex cognitive tasks engage outcome simulation and emotional motivation modulation (HUA et al., 2022; Pessiglione and Daunizeau, 2021). These functions align with OFC’s core roles in value computation and social signal decoding (Liao et al., 2024; Marciano et al., 2023). Empirical studies indicate that the OFC facilitates strategic decision-making by constructing predictive behavioral outcome models. FNIRS revealed significantly higher OFC hemodynamic activity during basketball compared to non-team-based modalities (e.g., Footbike), confirming its task-specific activation (Gorrell et al., 2022; Howard and Kahnt, 2021). Basketball players exhibit strengthened OFC strategic optimization networks through sustained training, manifested as task-dependent hemoglobin concentration activation in the OFC during Flanker task. This process facilitates irrelevant stimuli filtering and optimal decision selection via dynamic OFC-ventral striatum reward circuit interactions (Gardner et al., 2020).

In contrast, Footbike training is a closed-skill, individual sport. It is characterized by precise motor control and balance modulation (Chow and Ha, 2024). This training enhances PFC-mediated interference inhibition and motor execution circuits through repetitive practice. Consequently, it induces functional specificity in brain network reorganization. Behavioral data demonstrated significant improvements without concurrent OFC activation. Footbike’s biomechanical demands align with PFC functions—primarily optimizing balance maintenance and trajectory control (Monteiro et al., 2024). Its behavioral advantages reflect enhanced motor efficiency. OFC activation requires task—driven demands for complex decision-making and social cognition. In contrast, Footbike training-lacking such requirements—does not mobilize OFC engagement, with neuroadaptations instead focusing on PFC-dependent motor skill optimization.

This task-specific neural divergence reveals the “functional sculpting” effects of exercise on the brain. Basketball reinforces OFC-mediated strategic optimization networks through complex decision-making and social cognition. Conversely, Footbike training shapes PFC-dependent interference-resistant circuits via sustained attentional engagement. Basketball’s interventional advantages further manifest in its dual neural modulation mechanisms. Dynamic competitive environments in team sports enhance dopaminergic neurotransmission efficiency (Xiao et al., 2021). During critical moments (e.g., precise passes, successful scoring, defensive maneuvers), motor reward effects are amplified. This amplification occurs via mesocortical dopaminergic pathways, establishing a positive feedback loop (Wang J. et al., 2024). This mechanism not only activates the OFC to optimize decision inhibition, but also upregulates dopaminergic signaling to substitute addiction-related hedonic feedback through mesolimbic-cortical pathways (Michalowska-Sawczyn et al., 2025). Neuroimaging studies elucidate that long-term basketball training enhances OFC efficiency in encoding long-term reward value when exposed to addiction-related cues, while PFC-mediated cognitive control suppresses immediate temptation responses. This prefrontal-limbic functional synergy provides a robust neurobiological foundation for addiction cessation.

In summary, Footbike training significantly enhances interference inhibition in college students with IA. Basketball and Footbike differentially sculpt brain functional networks. Basketball reinforces strategic networks (e.g., OFC-mediated decision optimization). Conversely, Footbike shapes interference-resistant circuits (e.g., PFC-dependent cognitive control). Furthermore, basketball uniquely enhances dopaminergic neurotransmission efficiency, offering a neurobiological foundation for addiction cessation through reward system recalibration.

## 5 Conclusion

Footbike intervention significantly improves inhibitory control in college students with IA. Behavioral data demonstrate the most pronounced reduction in reaction times during response inhibition tasks and a marked decrease in conflict effects for interference inhibition—indicating effective enhancement of domain-specific cognitive inhibition through this training protocol.

Neuroimaging analyses reveal that Footbike training enhances neural excitability in the DLPFC and FPC, thereby optimizing cognitive control network efficiency—a mechanism strongly linked to improved response inhibition. Crucially, Footbike and basketball exhibit distinct OFC modulation patterns: Basketball induces selective OFC excitation through its socially open nature, whereas Footbike augments interference processing efficiency via circuit-specific neural adaptations, ultimately enhancing interference inhibition.

### 5.1 Limitations

The absence of long-term tracking precluded examination of sustainability and potential rebound effects for internet addiction—a critical gap given its chronic nature. Measurement constraints of inhibitory function (e.g., limited spatial sensitivity of fNIRS and task specificity in neurocognitive assessments) may affect subdomain detection reliability. Finally, the standardized exercise prescription ignored individual adaptability thresholds to fixed intensity-duration protocols, potentially diluting intervention efficacy across participants.

## Data Availability

The raw data supporting the conclusions of this article will be made available by the authors, without undue reservation.

## References

[ref1] Aivaz K. A. Teodorescu D. (2022). College students’ distractions from learning caused by multitasking in online vs. face-to-face classes: a case study at a public University in Romania. Int. J. Environ. Res. Public Health 19:11188. doi: 10.3390/ijerph191811188, PMID: 36141459 PMC9517392

[ref2] Arsoniadis G. Botonis P. Bogdanis G. C. Terzis G. Toubekis A. (2022). Acute and long-term effects of concurrent resistance and swimming training on swimming performance. Sports (Basel) 10:29. doi: 10.3390/sports10030029, PMID: 35324638 PMC8953612

[ref3] Beerten-Duijkers J. Vissers C. Rinck M. Egger J. (2021). Inhibitory control and craving in dual disorders and recurrent substance use. Preliminary findings. Front. Psych. 12:569817. doi: 10.3389/fpsyt.2021.569817, PMID: 33613336 PMC7886692

[ref4] Brini S. Boullosa D. Calleja-Gonzalez J. Ramirez-Campillo R. Nobari H. Castagna C.. (2023). Neuromuscular and balance adaptations following basketball-specific training programs based on combined drop jump and multidirectional repeated sprint versus multidirectional plyometric training. PLoS One 18:e283026. doi: 10.1371/journal.pone.0283026 PMC10016698 36921008

[ref5] Chang Y. S. Arefin M. S. You Y. L. Kuo L. C. Su F. C. Wu H. W.. (2020). Effect of novel Remodeled bicycle pedal training on balance performance in athletes with functional ankle instability. Front. Bioeng. Biotechnol. 8:600187. doi: 10.3389/fbioe.2020.600187, PMID: 33195176 PMC7642596

[ref6] Chen H. Dong G. Li K. (2023). Overview on brain function enhancement of internet addicts through exercise intervention: based on reward-execution-decision cycle. Front. Psych. 14:1094583. doi: 10.3389/fpsyt.2023.1094583, PMID: 36816421 PMC9933907

[ref7] Chen L. Du B. Li K. Li K. Hou T. Jia F.. (2024). The effect of tDCS on inhibitory control and its transfer effect on sustained attention in children with autism spectrum disorder: an fNIRS study. Brain Stimul. 17, 594–606. doi: 10.1016/j.brs.2024.04.019, PMID: 38697468

[ref8] Chen J. Li X. Zhang Q. Zhou Y. Wang R. Tian C.. (2020). Impulsivity and response inhibition related brain networks in adolescents with internet gaming disorder: a preliminary study utilizing resting-state fMRI. Front. Psych. 11:618319. doi: 10.3389/fpsyt.2020.618319, PMID: 33519558 PMC7843793

[ref9] Chen Y. Wang X. Zhou C. (2023). Effects of different exercise patterns on drug craving in female methamphetamine-dependent patients: evidence from behavior and fNIRS. Ment. Health Phys. Act. 25:100534. doi: 10.1016/j.mhpa.2023.100534

[ref10] Chow G. Ha S. (2024). Positive skill transfer in balance and speed control from balance bike to pedal bike in adults: a multiphase intervention study. PLoS One 19:e298142. doi: 10.1371/journal.pone.0298142 PMC10903920 38422110

[ref11] de Azambuja G. Jorge C. O. Gomes B. B. Lourenco H. R. Simabuco F. M. Oliveira-Fusaro M. (2021). Regular swimming exercise prevented the acute and persistent mechanical muscle hyperalgesia by modulation of macrophages phenotypes and inflammatory cytokines via PPARgamma receptors. Brain Behav. Immun. 95, 462–476. doi: 10.1016/j.bbi.2021.05.002 33964434

[ref12] Dopsaj M. Zuoziene I. J. Milic R. Cherepov E. Erlikh V. Masiulis N.. (2020). Body composition in international Sprint swimmers: are there any relations with performance? Int. J. Environ. Res. Public Health 17:9464. doi: 10.3390/ijerph17249464, PMID: 33348744 PMC7766121

[ref13] Evans S. A. James D. Rowlands D. Lee J. B. (2022). Variability of the Center of Mass in trained triathletes in running after cycling: a preliminary study conducted in a real-life setting. Front. Sports Active Living 4:852369. doi: 10.3389/fspor.2022.852369, PMID: 35734240 PMC9207334

[ref14] Fan H. Qi S. Huang G. Xu Z. (2021). Effect of acute aerobic exercise on inhibitory control of college students with smartphone addiction. Evid. Based Complement. Alternat. Med. 2021, 1–9. doi: 10.1155/2021/5530126, PMID: 34394381 PMC8360726

[ref15] Fan H. Qi S. Wang X. Liu C. (2019). Relationship between physical exercise volume and test anxiety among sports college students. Sports World 7, 64–65. doi: 10.16730/j.cnki.61-1019/g8.2019.07.039

[ref16] Faul F. Erdfelder E. Lang A. G. Buchner A. (2007). G*power 3: a flexible statistical power analysis program for the social, behavioral, and biomedical sciences. Behav. Res. Methods 39, 175–191. doi: 10.3758/bf03193146, PMID: 17695343

[ref17] Friedman N. P. Robbins T. W. (2022). The role of prefrontal cortex in cognitive control and executive function [Journal Article; Research Support, N.I.H., Extramural; Research Support, Non-U.S. Gov't; Review]. Neuropsychopharmacology 47, 72–89. doi: 10.1038/s41386-021-01132-0, PMID: 34408280 PMC8617292

[ref18] Friehs M. A. Frings C. Hartwigsen G. (2021). Effects of single-session transcranial direct current stimulation on reactive response inhibition. Neurosci. Biobehav. Rev. 128, 749–765. doi: 10.1016/j.neubiorev.2021.07.013, PMID: 34271027

[ref19] Fu H. Y. Wang J. Hu J. X. (2023). Influence of physical education on anxiety, depression, and self-esteem among college students. World J. Psychiatry 13, 1121–1132. doi: 10.5498/wjp.v13.i12.1121, PMID: 38186731 PMC10768485

[ref20] Gardner M. Sanchez D. Conroy J. C. Wikenheiser A. M. Zhou J. Schoenbaum G. (2020). Processing in lateral orbitofrontal cortex is required to estimate subjective preference during initial, but not established, economic choice. Neuron 108, 526–537. doi: 10.1016/j.neuron.2020.08.010 32888408 PMC7666073

[ref21] Gorrell S. Shott M. E. Frank G. (2022). Associations between aerobic exercise and dopamine-related reward-processing: informing a model of human exercise engagement [Journal Article; Research Support, N.I.H., Extramural]. Biol. Psychol. 171:108350. doi: 10.1016/j.biopsycho.2022.108350, PMID: 35561818 PMC9869713

[ref22] Howard J. D. Kahnt T. (2021). To be specific: the role of orbitofrontal cortex in signaling reward identity. Behav. Neurosci. 135, 210–217. doi: 10.1037/bne0000455, PMID: 33734730 PMC8224467

[ref23] Hua Y. Li M. Wang Q. Feng C. Zhang J. (2022). The role of left orbitofrontal cortex in selective attention during automatic emotion regulation: evidence from transcranial direct current stimulation. Acta Psychol. Sin. 52, 1048–1056. doi: 10.3724/SP.J.1041.2020.01048

[ref24] Jiang D. Xu G. (2022). Effects of chains squat training with different chain load ratio on the explosive strength of young basketball players' lower limbs. Front. Physiol. 13:979367. doi: 10.3389/fphys.2022.979367, PMID: 36105293 PMC9465379

[ref25] Jor A. Lai C. H. Khan M. J. He Y. Lam W. K. Winser S. J.. (2025). Effects of somatosensory-stimulating foot orthoses on postural balance in older adults: a computerized dynamic posturography analysis. Gait Posture 119, 189–196. doi: 10.1016/j.gaitpost.2025.03.016, PMID: 40147271

[ref26] Kang W. Hernandez S. P. Rahman M. S. Voigt K. Malvaso A. (2022). Inhibitory control development: a network neuroscience perspective. Front. Psychol. 13:651547. doi: 10.3389/fpsyg.2022.651547, PMID: 36300046 PMC9588931

[ref27] Kang W. Wang J. Malvaso A. (2021). Inhibitory control in aging: the compensation-related utilization of neural circuits hypothesis. Front. Aging Neurosci. 13:771885. doi: 10.3389/fnagi.2021.771885, PMID: 35967887 PMC9371469

[ref28] Kim S. W. Jung W. S. Kim J. W. Nam S. S. Park H. Y. (2021). Aerobic continuous and interval training under hypoxia enhances endurance exercise performance with hemodynamic and autonomic nervous system function in amateur male swimmers. Int. J. Environ. Res. Public Health 18:3944. doi: 10.3390/ijerph18083944, PMID: 33918616 PMC8068973

[ref29] Kraplin A. Scherbaum S. Kraft E. M. Rehbein F. Buhringer G. Goschke T.. (2020). The role of inhibitory control and decision-making in the course of internet gaming disorder. J. Behav. Addict. 9, 990–1001. doi: 10.1556/2006.2020.00076 33136066 PMC8969738

[ref30] Kwag E. Zijlstra W. (2022). Balance tasks requiring inhibitory control; a scoping review of studies in older adults [Journal Article; Scoping Review]. Gait Posture 93, 126–134. doi: 10.1016/j.gaitpost.2022.01.025, PMID: 35139472

[ref31] Li L. Wang H. Luo H. Zhang X. Zhang R. Li X. (2020). Interpersonal neural synchronization during cooperative behavior of basketball players: a fNIRS-based Hyperscanning study. Front. Hum. Neurosci. 14:169. doi: 10.3389/fnhum.2020.00169, PMID: 32733216 PMC7358650

[ref32] Li M. Zhang Y. Chen T. Du H. Deng K. (2025). Group cycling in urban environments: Analyzing visual attention and riding performance for enhanced road safety. Accid. Anal. Prev. 209:107804. doi: 10.1016/j.aap.2024.107804, PMID: 39426157

[ref33] Li Q. Zhao Y. Wang Y. Yang X. He Q. Cai H.. (2024). Comparative effectiveness of open and closed skill exercises on cognitive function in young adults: a fNIRS study. Sci. Rep. 14:21007. doi: 10.1038/s41598-024-70614-0, PMID: 39251657 PMC11385981

[ref34] Liao J. Li J. Qiu Y. Wu X. Liu B. Zhang L.. (2024). Dissociable contributions of the hippocampus and orbitofrontal cortex to representing task space in a social context. Cereb. Cortex 34:bhad447. doi: 10.1093/cercor/bhad447, PMID: 38011099 PMC10793565

[ref35] Liu Z. Wang Q. Sun W. Song Q. (2023). Balancing sensory inputs: somatosensory reweighting from proprioception to tactile sensation in maintaining postural stability among older adults with sensory deficits. Front. Public Health 11:1165010. doi: 10.3389/fpubh.2023.1165010, PMID: 37213635 PMC10194835

[ref36] Luo S. Soh K. G. Zhao Y. Soh K. L. Sun H. Nasiruddin N.. (2023). Effect of core training on athletic and skill performance of basketball players: a systematic review. PLoS One 18:e287379. doi: 10.1371/journal.pone.0287379 PMC10286970 37347733

[ref37] Ma C. Wang Y. Fu J. Zhao X. (2025). The impact of different types of academic stress on subcomponents of executive function in high school students of different grades. Acta Psychol. Sin. 57, 18–35. doi: 10.3724/SP.J.1041.2025.0018

[ref38] Macoun S. J. Schneider I. Bedir B. Sheehan J. Sung A. (2021). Pilot study of an attention and executive function cognitive intervention in children with autism Spectrum disorders. J. Autism Dev. Disord. 51, 2600–2610. doi: 10.1007/s10803-020-04723-w, PMID: 33029666

[ref39] Madinabeitia-Cabrera I. Alarcón-López F. Chirosa-Ríos L. J. Pelayo-Tejo I. Cárdenas-Vélez D. (2023). The cognitive benefits of basketball training compared to a combined endurance and resistance training regimen: a four-month intervention study. Sci. Rep. 13:11132. doi: 10.1038/s41598-023-32470-2, PMID: 37429866 PMC10333364

[ref40] Marciano D. Staveland B. R. Lin J. J. Saez I. Hsu M. Knight R. T. (2023). Electrophysiological signatures of inequity-dependent reward encoding in the human OFC. Cell Rep. 42:112865. doi: 10.1016/j.celrep.2023.112865, PMID: 37494185 PMC12199477

[ref41] Media Forum (2025). The 55th statistical report on China’s internet development released. Media Forum 8:121.

[ref42] Menon V. D'Esposito M. (2022). The role of PFC networks in cognitive control and executive function. Neuropsychopharmacology 47, 90–103. doi: 10.1038/s41386-021-01152-w, PMID: 34408276 PMC8616903

[ref43] Michalowska-Sawczyn M. Huminska-Lisowska K. Chmielowiec K. Chmielowiec J. Stronska-Pluta A. Suchanecka A.. (2025). Association analysis of the dopaminergic receptor 2 gene Tag1B rs1079597 and personality traits among a cohort of professional athletes. Biol. Sport 42, 35–43. doi: 10.5114/biolsport.2025.139470, PMID: 40182709 PMC11963129

[ref44] Mikneviciute G. Allaert J. Pulopulos M. M. De Raedt R. Kliegel M. Ballhausen N. (2023). Acute stress impacts reaction times in older but not in young adults in a flanker task. Sci. Rep. 13:17690. doi: 10.1038/s41598-023-44356-4, PMID: 37848597 PMC10582047

[ref45] Monteiro P. Marcori A. J. Da C. N. Monteiro R. Coelho D. B. Teixeira L. A. (2024). Cortical activity in body balance tasks as a function of motor and cognitive demands: a systematic review. Eur. J. Neurosci. 60, 6556–6587. doi: 10.1111/ejn.16574 39429043

[ref46] Muehlbauer T. (2021). Effects of balance training on static and dynamic balance performance in healthy children: role of training duration and volume. BMC. Res. Notes 14:465. doi: 10.1186/s13104-021-05873-5, PMID: 34949215 PMC8697465

[ref47] Nakutin S. N. Gutierrez G. (2019). Effect of physical activity on academic engagement and executive functioning in children with ASD. Sch. Psychol. Rev. 48, 177–184. doi: 10.17105/SPR-2017-0124.V48-2

[ref48] Niwa Y. Shimo K. Ohga S. Tokiwa Y. Hattori T. Matsubara T. (2022). Effects of exercise-induced hypoalgesia at different aerobic exercise intensities in healthy young adults. J. Pain Res. 15, 3615–3624. doi: 10.2147/JPR.S384306, PMID: 36419538 PMC9677918

[ref49] Pan J. Tang H. (2025). Age-related effects on dynamic postural stability and prefrontal cortex activation during precision fitting tasks. PeerJ 13:e18548. doi: 10.7717/peerj.18548, PMID: 39897502 PMC11786713

[ref50] Papalia G. F. Papalia R. Diaz B. L. Torre G. Zampogna B. Vasta S.. (2020). The effects of physical exercise on balance and prevention of falls in older people: a systematic review and meta-analysis. J. Clin. Med. 9:2595. doi: 10.3390/jcm9082595 32796528 PMC7466089

[ref51] Pessiglione M. Daunizeau J. (2021). Bridging across functional models: the OFC as a value-making neural network. Behav. Neurosci. 135, 277–290. doi: 10.1037/bne0000464, PMID: 34060880

[ref52] Rowley M. Warner J. Harper S. A. Beethe A. Z. Whelan R. Ruddy K. L.. (2022). A method to assess response inhibition during a balance recovery step. Gait Posture 95, 56–62. doi: 10.1016/j.gaitpost.2022.04.009, PMID: 35453084

[ref53] Rubega M. Formaggio E. Di Marco R. Bertuccelli M. Tortora S. Menegatti E.. (2021). Cortical correlates in upright dynamic and static balance in the elderly. Sci. Rep. 11:14132. doi: 10.1038/s41598-021-93556-3 34238987 PMC8266885

[ref54] Salehi M. Abbaspour Z. Molana A. Shahini N. (2022). Impulsivity, inhibition, and internet addiction in medical students of north of Iran. Front. Psych. 13:1002625. doi: 10.3389/fpsyt.2022.1002625, PMID: 36741579 PMC9892633

[ref55] Sandroff B. M. Hillman C. H. Benedict R. H. Motl R. W. (2016). Acute effects of varying intensities of treadmill walking exercise on inhibitory control in persons with multiple sclerosis: a pilot investigation. Physiol. Behav. 154, 20–27. doi: 10.1016/j.physbeh.2015.11.008, PMID: 26569451

[ref56] Sansare A. Harrington A. T. Wright H. Alesi J. Behboodi A. Verma K.. (2021). Aerobic responses to FES-assisted and volitional cycling in children with cerebral palsy. Sensors (Basel) 21:7590. doi: 10.3390/s21227590, PMID: 34833666 PMC8622737

[ref57] Sathe A. Shenoy S. Sathe P. K. (2024). Observation of cerebral cortex activation during static balance task in sporting and non-sporting individuals: a cross sectional fNIRS study. J. Bodyw. Mov. Ther. 40, 300–306. doi: 10.1016/j.jbmt.2024.04.012, PMID: 39593601

[ref58] Serra L. Petrosini L. Mandolesi L. Bonarota S. Balsamo F. Bozzali M.. (2022). Walking, running, swimming: an analysis of the effects of land and water aerobic exercises on cognitive functions and neural substrates. Int. J. Environ. Res. Public Health 19:16310. doi: 10.3390/ijerph192316310, PMID: 36498383 PMC9740550

[ref59] Shao X. He L. Liu Y. Fu Y. (2023). The effect of acute high-intensity interval training and tabata training on inhibitory control and cortical activation in young adults. Front. Neurosci. 17:1229307. doi: 10.3389/fnins.2023.1229307, PMID: 37781251 PMC10536150

[ref60] Shi H. Deng R. Wang F. (2025). Mediating effect of basic psychological needs and moderating effect of gratitude in stressful life events affected internet addiction among college students. Chin. J. Health Educ. 41, 29–33. doi: 10.16168/j.cnki.issn.1002-9982.2025.01.005

[ref61] Silva L. Doyenart R. Henrique S. P. Rodrigues W. Felipe L. J. Gomes K.. (2020). Swimming training improves mental health parameters, cognition and motor coordination in children with attention deficit hyperactivity disorder. Int. J. Environ. Health Res. 30, 584–592. doi: 10.1080/09603123.2019.1612041 31081373

[ref62] Sugiyama T. Maeo S. Kurihara T. Kanehisa H. Isaka T. (2021). Change of direction speed tests in basketball players: a brief review of test varieties and recent trends. Front. Sports Act. Living 3:645350. doi: 10.3389/fspor.2021.645350, PMID: 33997779 PMC8117963

[ref63] Symeonidou E. R. Esposito N. M. Reyes R. D. Ferris D. P. (2023). Practice walking on a treadmill-mounted balance beam modifies beam walking sacral movement and alters performance in other balance tasks. PLoS One 18:e283310. doi: 10.1371/journal.pone.0283310 PMC10270570 37319297

[ref64] Tsai C. L. Ju J. Chen Z. (2022). The mediating role of prosocial and antisocial behaviors between team trust and sport commitment in college basketball players. Eur. J. Sport Sci. 22, 1418–1425. doi: 10.1080/17461391.2021.1973571, PMID: 34463197

[ref65] van Moorselaar D. Slagter H. A. (2020). Inhibition in selective attention. Ann. N. Y. Acad. Sci. 1464, 204–221. doi: 10.1111/nyas.14304, PMID: 31951294 PMC7155061

[ref66] Vansteenkiste P. Van Hamme D. Veelaert P. Philippaerts R. Cardon G. Lenoir M. (2014). Cycling around a curve: the effect of cycling speed on steering and gaze behavior. PLoS One 9:e102792. doi: 10.1371/journal.pone.0102792, PMID: 25068380 PMC4113223

[ref67] Veliks V. Talents D. Fernate A. Evelis K. Kolesovs A. (2024). Neural activation patterns in open-skilled and closed-skilled athletes during motor response tasks: insights from ERP analysis. Front. Sports Act. Living 6:1476210. doi: 10.3389/fspor.2024.1476210, PMID: 39629223 PMC11611579

[ref68] Wang Y. Li X. (2025). A longitudinal study on the effect of aerobic exercise intervention on the inhibitory control in college students with internet addiction. Front. Hum. Neurosci. 19:1500399. doi: 10.3389/fnhum.2025.1500399, PMID: 40078486 PMC11896995

[ref69] Wang Y. Li K. Ge X. Cao Y. (2020). Training and transfer effects of response inhibition training with online feedback on adolescents' and adults' executive function. Acta Psychol. Sin. 52, 1212–1223. doi: 10.3724/SP.J.1041.2020.01212

[ref70] Wang Y. Lin Y. Ran Q. Cao N. Xia X. Tan X.. (2024). Dorsolateral prefrontal cortex to ipsilateral primary motor cortex intercortical interactions during inhibitory control enhance response inhibition in open-skill athletes. Sci. Rep. 14:24345. doi: 10.1038/s41598-024-75151-4, PMID: 39420010 PMC11487194

[ref71] Wang H. Tang W. Zhao Y. (2023). Acute effects of different exercise forms on executive function and the mechanism of cerebral hemodynamics in hospitalized T2DM patients: a within-subject study. Front. Public Health 11:1165892. doi: 10.3389/fpubh.2023.1165892, PMID: 37333536 PMC10270376

[ref72] Wang J. Xu X. Wu Q. Zhou C. Yang G. (2024). The mediating effect of subject well-being between physical activity and the internet addiction of college students in China during the COVID-19 pandemic: a cross-sectional study. Front. Public Health 12:1368199. doi: 10.3389/fpubh.2024.1368199, PMID: 38645442 PMC11026853

[ref73] Watanabe K. Yamaguchi Y. Fukuda W. Nakazawa S. Kenjo T. Nishiyama T. (2020). Neuromuscular activation pattern of lower extremity muscles during pedaling in cyclists with single amputation of leg and with two legs: a case study. BMC. Res. Notes 13:299. doi: 10.1186/s13104-020-05144-9, PMID: 32571389 PMC7310265

[ref74] Wu Y. Feng X. Ji M. (2024). Effects of emotional loss and cognitive loss on suppression control in college students with mobile phone dependent. Chin. Ment. Health J. 38, 271–276. doi: 10.3969/j.issn.1000-6729.2024.03.013

[ref75] Xiao T. Jiao C. Yao J. Yang L. Zhang Y. Liu S.. (2021). Effects of basketball and Baduanjin exercise interventions on problematic smartphone use and mental health among college students: a randomized controlled trial. Evid. Based Complement. Alternat. Med. 2021, 1–12. doi: 10.1155/2021/8880716, PMID: 33574886 PMC7864751

[ref76] Xu G. Zhou M. Chen Y. Song Q. Sun W. Wang J. (2024a). Brain activation during standing balance control in dual-task paradigm and its correlation among older adults with mild cognitive impairment: a fNIRS study. BMC Geriatr. 24:144. doi: 10.1186/s12877-024-04772-1, PMID: 38341561 PMC10859010

[ref77] Xu G. Zhou M. Wang J. Mao D. Sun W. (2024b). The effect of sensory manipulation on the static balance control and prefrontal cortex activation in older adults with mild cognitive impairment: a functional near-infrared spectroscopy (fNIRS) study. BMC Geriatr. 24:1020. doi: 10.1186/s12877-024-05624-8, PMID: 39702053 PMC11660590

[ref78] Yang C. Chen R. Chen X. Lu K. H. (2021). The efficiency of cooperative learning in physical education on the learning of action skills and learning motivation. Front. Psychol. 12:717528. doi: 10.3389/fpsyg.2021.717528, PMID: 34744877 PMC8564497

[ref79] Yin H. Chen A. Ma Z. Li X. Liu M. (2014). A follow-up study on two kinds of exercise intervention programs for children's executive functions. China Sport Sci. 34, 24–28. doi: 10.16469/j.css.2014.03.001

[ref80] Young K. S. (1998). Internet addiction: the emergence of a new clinical disorder. Cyber Psychol. Behav. 1, 237–244. doi: 10.1089/cpb.1998.1.237

[ref81] Yu Q. Herold F. Becker B. Klugah-Brown B. Zhang Y. Perrey S.. (2021). Cognitive benefits of exercise interventions: an fMRI activation likelihood estimation meta-analysis [Journal Article; Meta-Analysis; Review]. Brain Struct. Funct. 226, 601–619. doi: 10.1007/s00429-021-02247-2, PMID: 33675397

[ref82] Yun S. Guo Z. Li S. Jia S. Liu C. Wang X.. (2025). The effects of an 8-week taekwondo exercise intervention on inhibitory control in university students with depressive symptoms demonstrated the following-evidence from behavior and ERPs. BMC Psychiatry 25:169. doi: 10.1186/s12888-025-06598-6, PMID: 40001105 PMC11863427

[ref83] Zhang Y. Li G. Liu C. Chen H. Guo J. Shi Z. (2023a). Mixed comparison of interventions for different exercise types on students with internet addiction: a network meta-analysis. Front. Psychol. 14:1111195. doi: 10.3389/fpsyg.2023.1111195, PMID: 37303910 PMC10249056

[ref84] Zhang G. Yang L. Zhang J. Zhang J. (2023b). Impairment of inhibitory control in heroin abstinents: evidence from a dual inhibitory task. Chin. J. Clin. Psychol. 31, 300–304. doi: 10.16128/j.cnki.1005-3611.2023.02.008

[ref85] Zhang S. S. Zhong Y. Q. Li X. Peng M. (2024). Dissociation of prepotent response inhibition and interference control in problematic internet use: evidence from the go/no-go and flanker tasks. BMC Psychol. 12:216. doi: 10.1186/s40359-024-01698-6, PMID: 38637843 PMC11027223

[ref86] Zhao J. L. Jiang W. T. Wang X. Cai Z. D. Liu Z. H. Liu G. R. (2020). Exercise, brain plasticity, and depression. CNS Neurosci. Ther. 26, 885–895. doi: 10.1111/cns.13385, PMID: 32491278 PMC7415205

[ref87] Zhao Q. Liu J. Zhou C. Liu T. (2024). Effects of chronic aerobic exercise on attentional bias among women with methamphetamine addiction. Heliyon 10:e29847. doi: 10.1016/j.heliyon.2024.e29847, PMID: 38694043 PMC11058292

[ref88] Zheng K. Deng Z. Qian J. Chen Y. Li S. Huang T. (2022). Changes in working memory performance and cortical activity during acute aerobic exercise in young adults. Front. Behav. Neurosci. 16:884490. doi: 10.3389/fnbeh.2022.884490, PMID: 35983476 PMC9379142

[ref89] Zhou Z. Wan Y. Li C. Yuan J. Gao G. Cui H.. (2024). Effectiveness of sports intervention: a meta-analysis of the effects of different interventions on adolescent internet addiction. J. Affect. Disord. 365, 644–658. doi: 10.1016/j.jad.2024.08.064, PMID: 39147163

